# Leveraging Self-Affirmation to Improve Behavior Change: A Mobile Health App Experiment

**DOI:** 10.2196/mhealth.9151

**Published:** 2018-07-19

**Authors:** Aaron Springer, Anusha Venkatakrishnan, Shiwali Mohan, Lester Nelson, Michael Silva, Peter Pirolli

**Affiliations:** ^1^ Palo Alto Research Center Palo Alto, CA United States; ^2^ Computer Science Department University of California Santa Cruz Santa Cruz, CA United States

**Keywords:** mHealth, self-affirmation, behavior change, attrition, adherence, health behavior, telemedicine, treatment adherence and compliance

## Abstract

**Background:**

mHealth interventions can help to improve the physical well-being of participants. Unfortunately, mHealth interventions often have low adherence and high attrition. One possible way to increase adherence is instructing participants to complete self-affirmation exercises. Self-affirmation exercises have been effective in increasing many types of positive behaviors. However, self-affirmation exercises often involve extensive essay writing, a task that is not easy to complete on mobile platforms.

**Objective:**

This study aimed to adapt a self-affirmation exercise to a form better suited for delivery through a mobile app targeting healthy eating behaviors, and to test the effect of differing self-affirmation doses on adherence to behavior change goals over time.

**Methods:**

We examined how varied self-affirmation doses affected behavior change in an mHealth app targeting healthy eating that participants used for 28 days. We divided participants into the 4 total conditions using a 2×2 factorial design. The first independent variable was whether the participant received an initial self-affirmation exercise. The second independent variable was whether the participant received ongoing booster self-affirmations throughout the 28-day study. To examine possible mechanisms through which self-affirmation may cause positive behavior change, we analyzed three aspects of self-affirmation effects in our research. First, we analyzed how adherence was affected by self-affirmation exercises. Second, we analyzed whether self-affirmation exercises reduced attrition rates from the app. Third, we examined a model for self-affirmation behavior change.

**Results:**

Analysis of 3556 observations from 127 participants indicated that higher doses of self-affirmation resulted in improved adherence to mHealth intervention goals (coefficient 1.42, SE 0.71, *P*=.04). This increased adherence did not seem to translate to a decrease in participant attrition (*P* value range .61-.96), although our definition of attrition was conservative. Finally, we examined the mechanisms by which self-affirmation may have affected intentions of behavior change; we built a model of intention (*R*^2^=.39, *P*<.001), but self-affirmation did not directly affect final intentions (*P* value range .09-.93).

**Conclusions:**

Self-affirmations can successfully increase adherence to recommended diet and health goals in the context of an mHealth app. However, this increase in adherence does not seem to reduce overall attrition. The self-affirmation exercises we developed were simple to implement and had a low cost for both users and developers. While this study focused on an mHealth app for healthy eating, we recommend that other mHealth apps integrate similar self-affirmation exercises to examine effectiveness in other behaviors and contexts.

## Introduction

### Background

Millions of people are turning to mHealth apps to improve their physical and mental well-being. Over 50% of mobile phone users in the United States use mHealth apps; this rate doubled from 2014 to 2016 [1]. mHealth apps may offer effective low-cost solutions to major chronic health problems, which is especially attractive now, at a time when medical expenses in the United States have more than tripled in the past 50 years [2]. mHealth apps personalize medicine on a massive scale, therefore equipping patients to confront common problems such as smoking [3]. A review of mHealth interventions concluded that there is enough evidence to indicate that these interventions are effective, but more research should focus on integrating mHealth interventions into daily practice [4]. One of the primary problems with daily practice for mHealth interventions is that they are associated with poor adherence and attrition.

### Adherence and Attrition

Nearly 50% of people who started using an mHealth app at one point reported that they no longer used them [1]. Other interventions delivered remotely through the internet often have extremely high attrition rates [5].We must tackle these adherence and attrition problems so as to have a positive impact on users of mHealth apps.

Adherence and attrition are both problematic for mHealth systems, but it is important to distinguish between them. Adherence refers to the act of following the instructions for the app or intervention. For example, if an app sets a goal for a participant to eat 5 servings of fruits and vegetables, users are considered to have adhered if they have met that goal. We define attrition similarly to Eysenbach’s “nonusage attrition” [6]. Attrition refers to the population-level phenomenon of participants stopping use of an app and not returning as time goes on. This makes attrition a long-term product of poor adherence; improving one necessarily improves the other.

Attrition is not simply a fixed cost when delivering interventions electronically. Attrition can be studied, characterized, and reduced. In calling for a “science of attrition”, Eysenbach [6] laid out hypothetical proposed factors influencing attrition for further study. This call was answered with studies that examined attrition in Web-based interventions. Results were disheartening. At least two of these experiments [7,8] ended by concluding that, due to attrition, “…intervention programs may reach those who need them the least” [8]. In one intervention, participants who dropped out were interviewed to closely examine their motivation for discontinuing the program [9]. One major reason for attrition was that participants found the information threatening; they were not comfortable confronting their disease in such a manner. These problems must be overcome in order to have effective mHealth interventions. One solution may be psychological interventions called self-affirmation exercises.

### Self-Affirmation Exercises

An effective technique to increase adherence to recommended health behaviors is through the use of *self-affirmation exercises* [10-12]. Self-affirmation exercises are activities in which individuals focus on and affirm personally important values. For example, a participant who highly values their family would reflect on how their lives reflect this value or specific times when this value has influenced their behavior. Self-affirmation exercises have produced positive effects in many common health goals, including reducing smoking, reducing alcohol consumption, and increasing fruit and vegetable intake [12-17]. Self-affirmations can have long-term effects on behavior, spanning years in one deployment [15]. While the mechanism of self-affirmation’s effectiveness is debated, it seems to increase the likelihood that a participant will carefully consider information in a threatened realm; for example, self-affirmed smokers who read information about the deleterious effects of smoking may be less likely to outright reject the information [14]. These effects are present in other domains, including alcohol abuse [18], and in the domain of our experiment, healthy eating [19].

Unfortunately, self-affirmation exercises are often time-consuming writing prompts called values essays. These involve writing for up to 10 minutes about the importance of a single value such as friendships or family [20]. The extensive nature of the values essay makes it a poor fit for delivery through mobile phones. Writing an essay on a mobile phone is time consuming, and interventions that are time consuming may lead to participants not adhering to instructions [21]. Considering the effectiveness of self-affirmation and the increasing deliverance of interventions through mobile phones [22,23], there is a need to adapt self-affirmation exercises to the mobile medium that do not require long writing and reading tasks.

### Objectives

Methods other than self-affirmation have been tested to increase adherence and reduce attrition in mHealth interventions. Adding social support to an existing physical and mental well-being intervention was found to increase adherence [24]. An adaptive intervention that modulated exercise difficulty to user ability increased adherence when compared with statically scheduled controls [25]. While these interventions may be effective, they involve major restructuring of systems. Self-affirmation exercises like the ones we implemented could be easily added to many current systems.

We developed an mHealth app called Coach to enable users to record progress toward healthy eating goals. We focused on healthy eating because self-affirmations may decrease the difficulty of motivating participants to change behavior with distant consequences. Coach delivered the self-affirmation exercises that we created. To explore self-affirmation dosing, we compared different experimental groups that completed an extensive self-affirmation exercise initially with groups that continually self-affirmed throughout the study. We addressed the following questions:

- Can self-affirmation exercises be translated to mobile delivery to produce positive changes outside of controlled laboratory settings?
- Do higher doses of self-affirmation result in greater adherence than lower doses of self-affirmation?
- Does a higher dose of self-affirmation result in less attrition due to nonuse?
- What mechanisms mediate the effects of these self-affirmation exercises on behavior?

## Methods

### Intervention Groups

**Figure 1:**
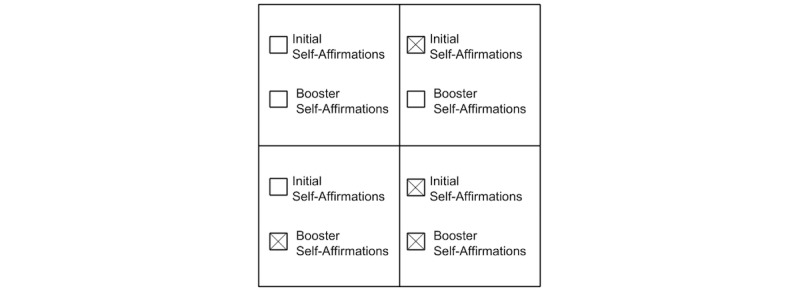
The 2x2 experimental design demonstrates the different independent variables across the groups.

To test our research questions, we developed and implemented the Coach app to record healthy eating behaviors and deliver our interventions. We randomly assigned participants to 4 groups, a 2 (initial self-affirmation vs control) × 2 (recurrent self-affirmation boosters vs control) factorial design (Figure 1).

In the groups receiving initial self-affirmations, participants completed a self-affirmation in the presurvey portion of the study. Groups receiving booster self-affirmations received small doses of self-affirmation exercises continually throughout the study. Thus, the initial and booster self-affirmations group received a high dose of self-affirmation through the study, while other conditions received lower doses of self-affirmation or none at all.

### Recruitment

We primarily recruited participants through online means, including Craigslist (Craigslist Inc, San Francisco, CA, USA), Nextdoor (Nextdoor Inc, San Francisco, CA, USA), and Reddit (Reddit Inc, San Francisco, CA, USA). Another source was an internal email list of a US West Coast research center. Participants were primarily located in the San Francisco Bay Area. Participant eligibility was determined by 3 factors: (1) age over 18 years, (2) available laptop or desktop computer and mobile phone running the Android or iOS operating systems, and (3) fruit and vegetable consumption below recommended levels (5 combined servings per day) [26].

Participants were compensated with a gift certificate for up to a maximum of US $50 for their participation. Compensation was prorated by amount of participation in the study. To receive full compensation, participants needed to complete the initial survey and the postsurvey, and make 20 out of 28 possible daily entries in the app.

### Ethical Approval

This study protocol, HSC-2016-04, was approved on July 13, 2016 by the institutional review board at Xerox PARC, Palo Alto, CA, USA. Participants completed informed consent forms as the very first step of the intake survey. The informed consent detailed the experiment, compensation, and the participant’s ability to withdraw from the study at any time for any reason.

### The Coach App System

**Figure 2:**
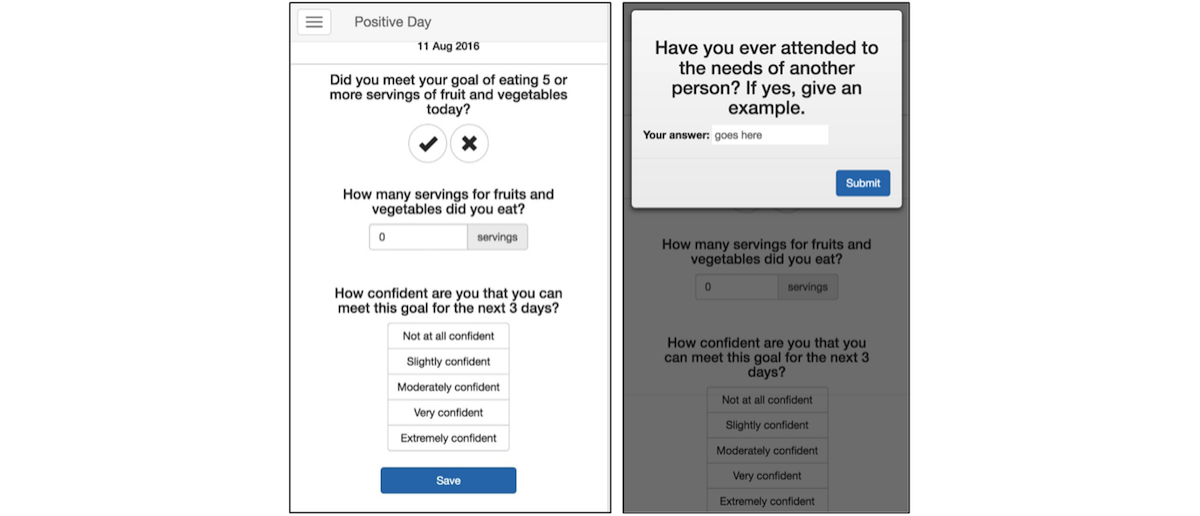
The Coach app homepage (left) and example question adapted from the kindness questionnaire (right).

We developed the Coach app specifically to study behavior change adherence and attrition in an mHealth setting. As such, we implemented only the most central features for reporting behavior and delivering interventions. This can be seen in the relatively unadorned interface in Figure 2. Any other features would only have obscured and confounded our primary research questions. In this experiment, the goal for participants was to consume 5 combined servings of fruit and vegetables per day.

Central to the Coach app is the reporting home page (Figure 2, left). Each day, the reporting page contained 2 primary questions about their progress toward consuming 5 servings of fruits and vegetables and 1 question about their confidence in continuing to meet this goal. This page saved the reported information and was updatable throughout the entire day. At the end of the day, the 3 questions were recorded in their final form and the page was cleared to enable reporting for the upcoming day.

Ancillary to the main reporting page were the exercises that were delivered once per week (on days 5, 12, 19, and 26) to participants in the booster conditions. Users received a push notification that there were questions for them to answer in the app. On tapping the notification or manually opening the app, they were greeted with a pop-up window that asked them questions. For the groups receiving self-affirmation boosters, the self-affirming questions were shown in this pop-up window, whereas controls were shown unrelated opinion questions. After answering these questions, participants were taken to the report page.

Two other screens were accessible in the app from the main drop-down menu. The first was an About screen that described the app and linked to the terms and condition of use. The second screen consisted of instructions on how to report within the app and instructions for general use.

### App Content

#### Self-Affirmation Initial Manipulation

Following Sherman et al [27], the initial affirmation was a standard values essay. First, participants rank ordered a list of 10 values, such as esthetic appreciation, relations with friends and family, and romantic values. Then participants wrote 3 reasons that their number 1–ranked value was important to them and wrote about a past experience where they demonstrated that value. Participants in the control condition were similarly asked to write about their last (10th)-ranked value and 3 reasons it could be important to someone else and how such a person might demonstrate that value.

#### Self-Affirmation Booster

In this work, we adapted a commonly used self-affirmation exercise [28] for use in the Coach app. The self-affirmation exercise we adapted is known as a kindness questionnaire. This kindness questionnaire is a set of 10 yes-no binary questions that participants answer and may elaborate on. The kindness questionnaire has been shown to have equal self-affirming effects to the longer values essays [29]. Previous self-affirmation interventions followed a model of extensive self-affirming, displaying threatening health information, and then evaluating participants [10]. We broke this mold to more closely examine the effects of self-affirmation timing and dose on health behavior change.

Rather than extensive self-affirming, we used multiple small self-affirmations we called *booster self-affirmations.* Booster self-affirmations were inspired by similar previous works that administered self-affirmations repeatedly over the course of longer-term experiments [15,30]. However, these experiments used the original time-consuming self-affirmation exercises that may not integrate easily into mHealth apps. A previous study suggested that there is no minimum level of engagement with self-affirmation exercises to gain their positive effects [13], which gave us room to adapt these exercises. To lessen the writing burden and make self-affirmations less time consuming on mobile phones, we adapted the kindness questionnaire [28] to a form more suitable for mobile devices.

Rather than showing the full 10 questions to the participants during a booster affirmation, we simply showed them 2 questions for each affirmation booster. Figure 2 (right) shows an example question adapted from the kindness questionnaire. We adapted questions so participants could include both the binary answer and an example in the given text box [13]. These questions from the kindness survey were specifically constructed so that nearly everyone would answer affirmatively [28]. Participants in the control conditions for the boosters received similarly adapted questions from the control manipulation (asking non–self-affirming questions) to ensure that control participants received the same number of notifications and spent a similar amount of time in the app [28].

#### Manipulation Check

The manipulation check immediately followed the initial self-affirmation manipulation or control manipulation. The manipulation check consisted of a 3-item scale to assess the degree of self-affirmation that participants felt [31]. It consisted of 3 questions starting with “The task on values made me think about things,” and participants answered on scales of “Things I don’t like about myself” to “Things I like about myself;” “Things I’m bad at” to “Things I’m good at;” and “Things I don’t value about myself” to “Things I value about myself.”

#### Threatening Health Information

Following the manipulation check, participants were shown a document outlining the risks of not consuming enough fruit and vegetables. This is standard practice for self-affirmation interventions that attempt to improve health behaviors [10,14,19,28]. The self-affirmation seems to allow participants to better accept health information that may be threatening [18]. The threatening health information document was based on a webpage created by the UK National Health Service (NHS) [26]. These NHS recommendations mirrored United States Department of Agriculture (USDA) recommendations in [32], and the text we used was not labeled as being from the NHS. We chose the NHS text because it was more succinct than comparable sources from the USDA. We slightly modified the text to better highlight significant health threats attributed to not consuming enough fruits and vegetables, including obesity, cancer, high blood pressure, stroke, and diabetes.

#### Extended Parallel Process Model Measure

We examined participants’ responses to our self-affirmations and threatening stimuli in the context of the extended parallel process model (EPPM). The EPPM explains the effects of fear appeals on intentions and behavior change [33]. Recall that self-affirmation exercises often follow the outline of a fear appeal: the participant self-affirms, then they are shown threatening health information. Recent work exploring self-affirmation theory and the EPPM [12] found that self-affirmation contributed to explained variance of intentions to change consumption of fruits and vegetables but did not examine the resulting behavior. Unfortunately, Napper et al [12] ignored fear as a first-class model parameter. According to Witte [33], the entire point of the EPPM is “putting the fear back into fear appeals.”

We measured the major EPPM constructs: self-efficacy, response efficacy, threat, and intention. We also measured fear responses via the Positive and Negative Affect Schedule with added measures to form a fear subscale [34]. These measures, excluding fear, were identical to the measures used in a previous study examining the EPPM and self-affirmation [12]. The measured constructs were threat, efficacy, and intentions. Threat was measured by 1 severity item (“How serious are the health consequences of not eating at least 5 portions of fruit and vegetables each day?”) and 2 susceptibility items (“My chances of experiencing heart disease or some cancers in the future if I do not eat at least 5 portions of fruit and vegetables each day are...” and “How likely is it that you will experience poor health in the future if you do not eat at least 5 portions of fruit or vegetables each day?”). Efficacy was measured by 2 self-efficacy items (“I know for sure that I could adhere to eating at least 5 fruit and vegetables each day if I really wanted to” and “If I were to eat at least 5 portions of fruit and vegetables each day I would reduce my risk of heart disease and some cancers”) and 1 response-efficacy item (“Eating at least 5 portions of fruit and vegetables each day will reduce my risk of heart disease and some cancers”). Previous studies indicated that these measures are internally consistent; the Cronbach alpha of the combined threat measure was .77, and the combined efficacy measure was .78 [12]. Use of self-affirmation in health-related realms follows the model of a fear appeal; thus, the EPPM should provide us with information about which variables in the model are affected by self-affirmation.

### Statistical Analysis

First, we examined adherence. Our longitudinal data consisted of users tracking whether they successfully met their fruit and vegetable consumption goal each day. Given that this is a binary response variable, we modeled it using a logistic regression. However, individuals may have initial differences and there may also be temporal differences in how users respond over the course of the 28-day study. To address these differences, we used a mixed-effects logistic regression that allowed us to control for the temporal and individual differences and carefully examine the fixed differences of the initial self-affirmation and self-affirmation boosters.

Second, we examined attrition. We used a Kaplan-Meier survival curve to visually examine the full cohort’s survival curve. We then fitted a Cox proportional hazards model to examine whether the self-affirmation conditions had an effect on not just adherence, but overall attrition from the app.

Third, we examined the mechanisms behind self-affirmation using the EPPM. This analysis used a linear regression model to examine how different factors interacted with the self-affirmation to influence user intentions.

We calculated the number of participants needed for this analysis using Diggle’s longitudinal power analysis [35]. We specified a significance level of .05, power of .80, n=28 repeated measures, and a conservative repeated measures correlation of .6. We used Fotuhi’s effect size of 0.24 [36], which was the closest experimental design to ours that used self-affirmation in a health behavior context. This calculation resulted in requiring 132 participants for a fully powered experiment.

## Results

### Sample Characteristics

Recruitment resulted in 134 participants completing the intake survey. Among these, 127 downloaded and signed up within the Coach mobile app. Of these 127 participants, 90 identified as female, 36 identified as male, and 1 identified as nonbinary. Participants reported averaging 2.23 servings of fruits and vegetables the day before filling out the intake survey. To confirm that no group differences existed at intake, we tested baseline group differences in possible confounds. No differences existed in age (*P*=.24), sex (*P*=.79), ethnicity (*P*=.89), body mass index (*P*=.50), or prior average intake of fruits and vegetables (*P*=.26). There was no discernable difference in the manipulation check between initial conditions (*t*_119.08_=–0.34, *P*=.74).

### Self-Affirmation and Behavior Change

**Figure 3:**
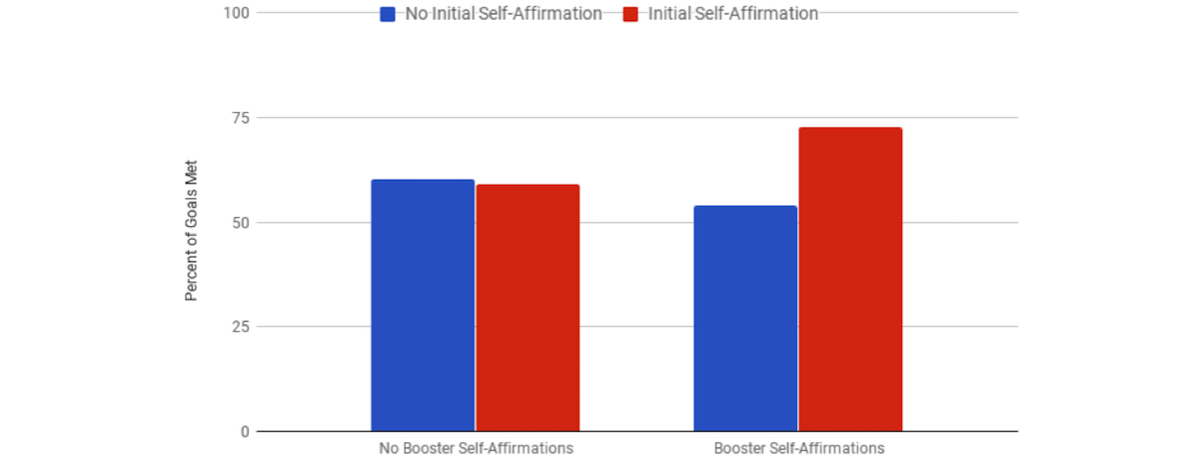
Percentage of goals met by condition.

Our first research question concerned whether a higher dose of self-affirmation would increase goal adherence for our participants. This means that the group receiving both the initial affirmation and affirmation boosters would outperform the other conditions in meeting their daily goals of fruit and vegetable consumption. We tested this hypothesis using a mixed-effects logistic regression model. The model was specified with random effects to control for participant differences and temporal differences. Condition independent variables (initial affirmation and booster affirmation) were specified as fixed effects with an interaction. We found that participants who received both the initial affirmation and booster affirmations were significantly more likely (*P*=.04), to meet their goals of fruit and vegetable consumption throughout the study. Higher coefficients in Table 1 indicate higher log odds of a participant in each condition reporting they met their fruit and vegetable intake goal. As Figure 3 shows, this resulted in overall higher probability of adherence in the group receiving the highest dose of self-affirmation exercises.

**Table 1:** Coefficients for the logistic regression model of goal adherence.

Coefficients	Coefficient estimate	SE	*z* value	*P* value
(Intercept)	0.56	0.35	1.61	.11
Initial self-affirmation	–0.12	0.50	–0.25	.81
Self-affirmation boosters	–0.38	0.50	–0.76	.45
Initial * boosters	1.42	0.71	2.01	.04^a^

^a^Significant at *P*<.05.

### Self-Affirmation and Attrition

Our second research question concerned the relationships of self-affirmation dose and attrition. We examined attrition using the Kaplan-Meier survival curve and Cox proportional hazards model. We defined a user to have dropped out of the study after they had missed 5 consecutive daily entries. While this may be conservative, we arrived at this number by examining the variance in total times reported compared with the longest streak of consecutive misses from the participants. We noted that variance was generally low for participants with miss streaks between 1 and 4, indicating that they generally went on to complete the remaining study. However, around a miss streak of 5, the variance grew dramatically, indicating that participants who missed 5 entries in a row became more likely to drop out and never return to the study. This method is analogous to using the well-established scree test for determining the number of factors in a factor analysis [37]. For the following attrition analyses, we defined the time of dropout as the first day in a string of 5 or more consecutive nonreports from a single participant.

**Figure 4:**
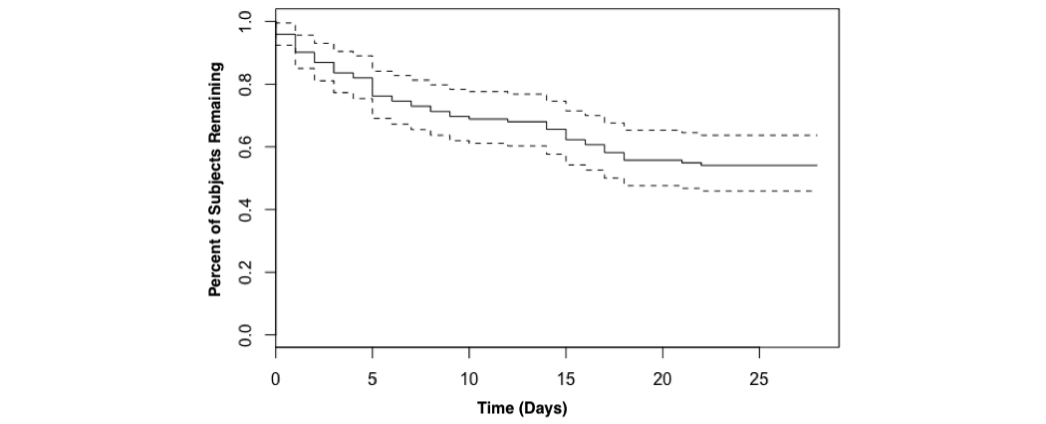
“Survival” of Coach app participants as shown by a Kaplan-Meier survival curve with 95% confidence intervals (dashed lines).

Figure 4 shows the full cohort’s attrition curve with 95% confidence intervals. This was calculated using a Kaplan-Meier estimator with right-censored survival data. Right-censored data fitted our data because many participants “survived” until the end of the experiment; therefore, we don’t truly know how long they would have continued reporting after the experiment finished. The survival curve generally follows what has been found previously for eHealth app adoption. The curve is fairly steep at the beginning and then gradually flattens to a core group of users over time [6].

To test for differences in survival between conditions, we calculated a Cox proportional hazards model. We specified the proportional hazards dropout using EPPM variables (intentions, threat, response efficacy, self-efficacy, and fear) and the condition. This model using EPPM variables and condition was significant at *P*=.04. Table 2 shows the coefficients of this model.

### Mechanism of Self-Affirmation’s Effects

Our final research question concerned what mechanisms mediate the effects of self-affirmation. To examine this, we measured and modeled the EPPM in order to see which factors of the model were modified by the initial self-affirmation exercises.

We expected that including the initial self-affirmation as a predictor in the EPPM model would increase the explained variance in intentions of the model. Consistency between subscale items in the EPPM questions was high (alpha range .80-.89) and thus we averaged the subscales to create single scores for each subitem. We specified a linear regression in the form of Napper et al [12] with the addition of the averaged fear subscale (Table 3 shows the coefficients). Given that we included fear as a primary variable, we specified fear and an interaction between self-efficacy and fear within the model in accordance with the original conception of the danger control process [33]. We found strong support for the EPPM model as a whole (*R*^2^=.39, *P*<.001), but the addition of initial affirmation and associated interactions as predictors did not improve the explanatory power of the model. Table 3 shows the final EPPM model.

**Table 2:** Coefficients for the Cox proportional hazards model.

Coefficients	Coefficient estimate	SE	*z* value	*P* value
Initial self-affirmation	–0.02	0.43	–0.05	.96
Self-affirmation boosters	0.18	0.41	0.43	.67
Self-efficacy	0.19	0.31	0.60	.55
Response efficacy	–0.05	0.35	–0.14	.89
Intentions	0.10	0.31	0.33	.74
Fear	0.17	0.05	3.69	<.001^a^
Initial * boosters	0.28	0.56	0.50	.61

^a^Significant at *P*<.05.

**Table 3:** Coefficients for the extended parallel process model linear regression model.

Coefficients	Coefficient estimate	SE	*z* value	*P* value
(Intercept)	1.52	1.17	1.31	.19
Threat	–0.45	0.36	-1.26	.21
Self-efficacy	0.05	0.39	0.12	.89
Response efficacy	0.42	0.10	4.29	<.001^a^
Fear	0.23	0.09	2.64	<.001^a^
Initial self-affirmation	0.59	0.53	1.13	.26
Threat * self-efficacy	0.16	0.11	1.48	.14
Fear * self-efficacy	–0.08	0.03	-3.01	<.001^a^
Fear * initial	0.04	0.03	1.12	.27
Threat * initial	0.01	0.13	0.09	.93
Self-efficacy * initial	–0.25	0.15	-1.70	.09

^a^Significant at *P*<.05.

## Discussion

### Principal Results

We demonstrated that continual self-affirmation exercises resulted in increased adherence among participants using an mHealth app targeting increasing fruit and vegetable consumption. However, our work also demonstrated the difficulty in adapting traditional laboratory-based interventions to the unstructured lives of mobile users. Traditional interventions for self-affirmation are relatively unstructured but are supported by the consistency of the laboratory environments in which they are administered. These results indicate that carefully constructed self-affirmation exercises can be integrated into unstructured daily life through mHealth systems and may improve adherence as a result.

### Self-Affirmation Promotes Adherence

We demonstrated significant positive effects on adherence for the experimental group that engaged in higher doses of self-affirmations. The group receiving an initial self-affirmation and booster self-affirmations met their daily goals 21% more than the control (72.8 [317/435] and 60.4 [306/506], respectively, where numbers in brackets show how many goals the participants met over the records the participants made of their healthy eating where they didn't meet their goals; see Figure 2); this effect is large enough to have major effects in real-world scenarios.

Perhaps even more important, we demonstrated that this effect can be achieved in the context of an mHealth app. To our knowledge, no previous work on self-affirmation has used mHealth apps. mHealth apps present problems for typical self-affirmation exercises, including constantly changing environments, competing attentional requirements, and different affordances for inputs. This work indicated that self-affirmation exercises can be adapted in ways that make them amenable to increasing adherence in mHealth contexts. In turn, this enables these increases in adherence to benefit large groups of people, since mHealth apps have the ability to be deployed to a large number of people quickly.

### Self-Affirmation Exhibits Dosing Effects

We supported our primary hypothesis that repeated self-affirmation promotes goal-achieving behaviors in the context of an mHealth system designed to increase fruit and vegetable consumption. Interestingly, we seemed to have found a dose effect in the administration of self-affirmation exercises. Previous studies had increased doses of self-affirmation [15,30], but did not closely examine these effects. Our work may point toward a dose effect. The only group that significantly differed was the group that received both the initial and booster affirmations, the highest overall dose. This contrasts with previous hypotheses from Steele, the original author of the self-affirmation theory; as Steele [38] wrote: “there is no evidence yet to suggest that a minimum level of engagement with the manipulations is required before an individual is sufficiently affirmed.” Our work challenges this assumption and calls for further investigation into the dose-dependent effects of self-affirmation.

Prior experiments recording participant behavior after a single self-affirmation exercise have been mixed in showing beneficial effects. Some studies demonstrated that participants made beneficial behavioral changes in the weeks and months after self-affirming [13,14,19]. Others showed that, while participants’ initial attitudes and processing changed, their behavior was not affected by a single self-affirmation [18,28,39]. Similar to many of these studies, our group receiving only an initial self-affirmation did not show significant behavioral changes. Additionally, our initial self-affirmation group failed its manipulation check to differentiate it from the conditions that did not complete the initial self-affirmation. It is possible that previous studies and this study found no differences for a single self-affirmation because these doses of self-affirmation simply were not high enough to elicit changes. Another possibility is that the instability of administering these self-affirmation exercises outside of a laboratory setting increased the threshold of the dose needed.

An alternative interpretation of our results could be that, rather than exhibiting dosing effects, different self-affirmation exercises have varying effectiveness outside of laboratory settings. The values essay that served as our initial self-affirmation was relatively unstructured; participants wrote freely in answering a prompt with a few questions. The self-affirmations that we constructed based on the kindness questionnaire were more structured; we asked participants specific targeted questions and they then answered with an example from their lives. We cannot confirm that more structured self-affirmation exercises are more effective outside of laboratory settings, but this should be explored further. However, previous results indicated that unstructured self-affirmation exercises such as a values essay may be effective in Web-based contexts [30].

### Mechanism of Self-Affirmation

In agreement with previous work, the EPPM predicted intentions to change behavior (*R*^2^=.39). However, unlike in previous work by Napper et al [12], in our study, the self-affirmation condition added no explanatory effect. We measured the EPPM factors only after the initial self-affirmation in groups that received it; if we had repeated this measure at the end of the study following the higher doses, it is possible that we would have seen the effects that we hypothesized.

We expected to see an interaction between self-affirmation and self-efficacy but did not find that. Self-efficacy and self-affirmation have shown strong interactions in previous studies. These studies showed that self-affirmation benefitted those who were most at risk and felt that they did not have self-resources available to make the behavior changes required. Again, we expect that the lack of interaction between self-affirmation and self-efficacy in our data was due to the small effect of the initial affirmation.

In addition, we expected that fear levels would predict intentions. The EPPM indicates that fear affects intentions only as mediated by perceived threat [33,40]. However, this experiment indicated that fear influences intentions directly, as well as in interaction with self-efficacy. In a study by Popova [40], high perceived efficacy and presence of fear influenced danger control outcomes (high intentions), although mediated by perceived threat. Our results, however, do not support this hypothesis. First, in our model perceived threat did not mediate the relationship between fear or self-efficacy and intentions. Second, we found the opposite interaction between fear or self-efficacy and intentions. We found that, at high levels of self-efficacy, higher levels of fear actually corresponded with decreased intentions. At lower levels of efficacy, fear corresponded to higher intentions.

### mHealth System Recommendations

We found support for the inclusion of self-affirmation into systems promoting behavior change. We delivered self-affirmation in a way that is more amenable to mobile apps and resulted in improved adherence to health goals, thereby increasing health behaviors. Our results indicate that, in mobile environments, the dose of self-affirmation may matter greatly. This may be due to the changing environment when administering such interventions in natural settings.

As we have demonstrated, this form of self-affirmation is effective for behavior change in mHealth apps designed to improve user eating habits. Other studies have supported the effectiveness of more manual self-affirmation interventions with targets such as education [11], well-being and happiness [30], physical activity [41], smoking cessation [14], and reducing alcohol consumption [13]. Many mobile apps already exist to help support people in these realms, but none, to our knowledge, have integrated self-affirmation to increase this support. Self-affirmation could play a valuable role in increasing the effectiveness of these apps, particularly for users who may be most at risk.

### Limitations

This study had a few limitations that should be acknowledged when considering the results. Our study was underpowered. Our power analysis showed that we required 132 participants to be fully powered; due to 7 dropouts between the intake survey and app download, we analyzed only 127 participants. Additionally, 70.9% (90/127) of our sample identified as female; this could limit transferability to larger populations. It is also possible that our compensation strategy for participants influenced their adherence and attrition. While participants across conditions were all paid the same, it is possible that there was some interaction between compensation and self-affirmation that we cannot discern with this design.

### Future Work

We call for other systems researchers to examine their own mobile apps with an eye toward integrating these self-affirmation exercises to enhance their systems. While we demonstrated positive effects from self-affirmation in the context of healthy eating, prior non-mHealth experiments indicated broad applicability of mHealth to different behavior change targets. Self-affirmation has the potential to be the digital analog to aspirin: an intervention that has positive effects on behavior in many apps. Our self-affirmation exercises are simple to implement and require only a short time commitment. Translating these interventions to the mobile world has created a highly scalable and customizable technique to enhance behavior change.

This work raises a question of correct self-affirmation dose and response thresholds. This work indicates that higher doses of self-affirmation exercises may be effective in increasing goal adherence. Future work could explore the effectiveness of various self-affirmation schedules in augmenting existing mHealth apps with appropriate self-affirmation dosing schedules.

## Abbreviations

EPPM: extended parallel process model
NHS: National Health Service
USDA: United States Department of Agriculture
